# Multi-wavelength optical information processing with deep reinforcement learning

**DOI:** 10.1038/s41377-025-01846-6

**Published:** 2025-04-15

**Authors:** Qiuquan Yan, Hao Ouyang, Zilong Tao, Meili Shen, Shiyin Du, Jun Zhang, Hengzhu Liu, Hao Hao, Tian Jiang

**Affiliations:** 1 College of Computer Science and Technology, National University of Defense Technology, Changsha, China; 2 Institute for Quantum Science and Technology, College of Science, National University of Defense Technology, Changsha, China; 3 National Innovation Institute of Defense Technology, Academy of Military Science PLA, Beijing, China; 4 College of Advanced Interdisciplinary Studies, National University of Defense Technology, Changsha, China; 5 Hunan Research Center of the Basic Discipline for Physical States, National University of Defense Technology, Changsha, China

**Keywords:** Frequency combs, Integrated optics, Microwave photonics

## Abstract

Multi-wavelength optical information processing systems are commonly utilized in optical neural networks and broadband signal processing. However, their effectiveness is often compromised by frequency-selective responses caused by fabrication, transmission, and environmental factors. To mitigate these issues, this study introduces a deep reinforcement learning calibration (DRC) method inspired by the deep deterministic policy gradient training strategy. This method continuously and autonomously learns from the system, effectively accumulating experiential knowledge for calibration strategies and demonstrating superior adaptability compared to traditional methods. In systems based on dispersion compensating fiber, micro-ring resonator array, and Mach-Zehnder interferometer array that use multi-wavelength optical carriers as the light source, the DRC method enables the completion of the corresponding signal processing functions within 21 iterations. This method provides efficient and accurate control, making it suitable for applications such as optical convolution computation acceleration, microwave photonic signal processing, and optical network routing.

## Abstract (web summary)

Implementation of deep reinforcement learning-based calibration algorithm in multi-wavelength optical information processing systems based on dispersion compensating fiber, microring resonators, and Mach-Zehnder interferometers.

## Graphical abstract

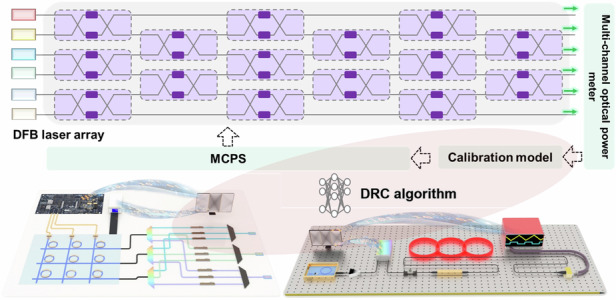

## Introduction

Optical information processing systems have emerged as essential components in numerous applications such as accelerating computing and broadband signal processing^1–3^. In particular, multi-wavelength optical information processing systems (hereafter multi-wavelength systems) capitalize on the intrinsic physical properties of light and wavelength division multiplexing^4^. These systems provide noteworthy advantages, such as high bandwidth, low attenuation, resilience to electromagnetic interference, and low energy consumption^5–7^. With photonic integrated circuits, the information processing capacity of multi-wavelength systems can be further enhanced, simultaneously reducing Size, Weight, and Power consumption (SWaP)^8^. There is a growing number of such systems across the realms of microwave photonics^9^ and optical computing^10–12^.

Nonetheless, the frequency-selective response may affect the efficacy and dependability of multi-wavelength systems in practical applications. For example, in dispersive compensating fiber (DCF)-based optical computing systems, non-uniform intensity distribution of the optical frequency comb and differences in device frequency responses across wavelengths can introduce computational errors; in Micro-Ring Resonator (MRR)-based systems, the resonance peak position and amplitude often deviate from theoretical predictions; and in Mach-Zehnder interferometer (MZI)-based systems, variations in waveguide responses across different wavelengths lead to performance discrepancies. These phenomena can be ascribed to various factors. First, fabrication processing constraints may result in device defects, causing the frequency response to deviate from the expected design specifications^13^. Second, the inherent non-uniformity of the frequency response originating from the gain medium could potentially affect the entire frequency response of the system^14^. Finally, the physicochemical characteristics of the operational environment might have an impact on the reliability of the system^15^.

Within the realm of electronic information and communication, a variety of calibration techniques have been applied to address frequency-selective response challenges^16–18^. One notable example is the introduction of a digital differentiator polynomial model by Xiang et al. to counteract the frequency-selective response in a time-interleaved Analog-to-Digital Converter (ADC)^19^. Another significant contribution comes from Zhou et al., who developed a lookup table-based calibration algorithm for Radio over Fiber^20^. Both methods collectively fall under the category of model-based calibration, which highly depends on intricate and rigorous modeling, thereby hindering their widespread adoption.

To enhance usability, researchers have focused on model-free calibration techniques rooted in optimization algorithms^21,22^, such as artificial neural networks for calibration in optical neural network^23^. However, there are still some limitations, as they often involve time-consuming iterations and exhibit sensitivity to environmental conditions, ultimately constraining their overall efficacy.

To reduce the errors caused by frequency-selective response in multi-wavelength systems while maintaining accuracy, usability, and effectiveness, this work presents the Deep Reinforcement learning-based Calibration (DRC). The DRC method incorporates training strategies from deep deterministic policy gradient (DDPG)^24,25^ to facilitate rapid adaptability for multi-wavelength systems. During the process of inferring calibration strategies, the DRC model undergoes continuous training and optimization. As a result, the calibration network accumulates calibration experience over time. Such a mechanism also enables the method to adapt to environmental disturbances such as vibration, humidity, and temperature fluctuations, provided that their amplitude remains moderate and the disturbance frequency is far below the convergence time of the calibration algorithm. Compared to traditional methods, this accumulation of experience significantly reduces the number of iterations required by DRC. The principles of the DRC method are described in Section 4. By leveraging reinforcement learning algorithms, DRC acquires knowledge through environmental interactions, dynamically and rapidly refining calibration strategies to bolster robustness against environmental perturbations^26–29^.

Moreover, this study involved an experimental validation process on three types of multi-wavelength systems which are utilizing DCF, MRR array, and MZI array, respectively. Each of these systems is designed for specific classification tasks, including the classification of the MNIST dataset^30^ and the Urbansound8K dataset^31^. In terms of model size, the DCF system employs 20 optical combs for convolution operations, the MRR system utilizes 9 micro-rings for multiply-accumulate (MAC) operations, and the MZI system includes 15 MZIs for MAC operations. The empirical results demonstrated that the DRC reached convergence within 21 iterations. Specifically, in the DCF-based system, the error of the output decreased by 78.0%. In the MRR array-based system, the variance in output among different MRRs dropped to 3.89 × 10^−4^. In the MZI array-based system, the relative output error after calibration by DRC was reduced by 85.4% compared to the results obtained prior to calibration using the standard voltage configuration. This work compares DRC to three other calibration techniques employing Genetic Algorithm (GA), Stochastic Parallel Gradient Descent (SPGD), and Proportional-Integral-Derivative (PID) algorithm respectively. Among these methods, DRC proved to be superior in terms of calibration efficiency and accuracy. This finding highlights the potential of DRC as an effective solution to the frequency-selective response challenges, which plays a key role in the precision and reliability improvement of multi-wavelength systems.

## Results

### Calibration of DCF-based System

**Fig. 1:**
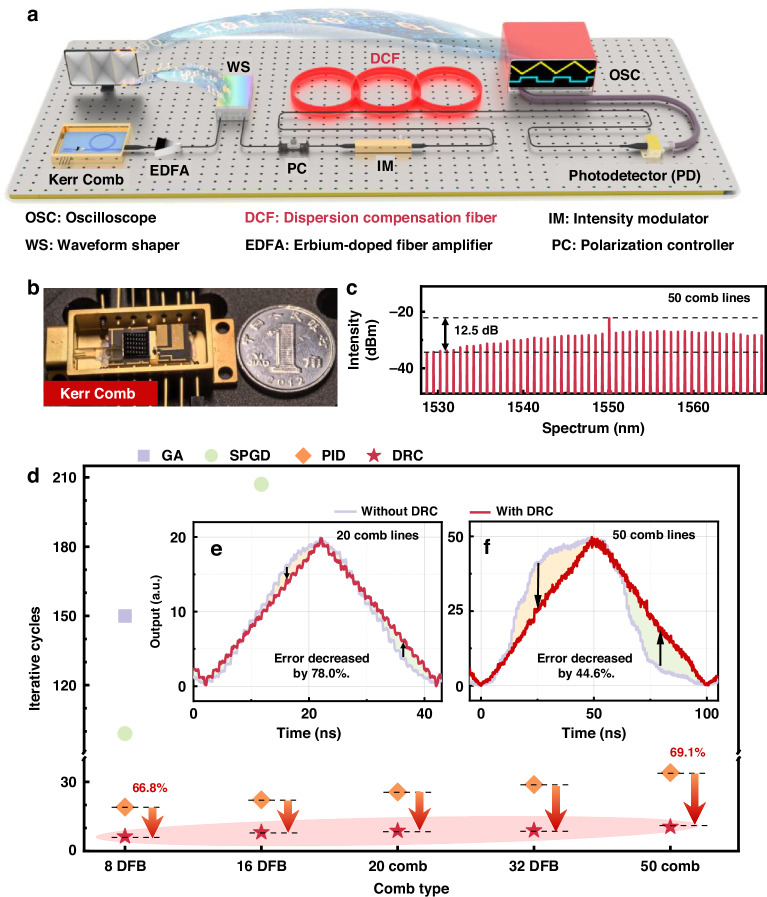
Calibration architecture and results for DCF-based system. **a** depicts the calibration link. **b** presents a photograph of the encapsulated chip of the Kerr comb. **c** displays the 50 optical comb lines used in the system, which were generated from the Kerr optical comb. **d** shows the iterative cycles to the 4 calibration algorithms when using laser sources with different numbers of comb lines. **e** and **f**, respectively present the comparative outcomes of the DCF-based system output with and without DRC calibration, using 20 comb lines and 50 comb lines as laser sources

Figure 1a showcases the DCF-based system utilizing Kerr Optical Frequency Comb (OFC)^32,33^. As shown in Fig. 1b, a photograph of the packaged laser source chip is provided. Due to bandwidth constraints of the Waveform Shaper (WS) used in this study, 20 and 50 comb lines are selected to test the calibration capability of the DRC. The spectrum of 50 comb lines is presented in Fig. 1c. The working principle and chip details of this system can be found in Supplementary Note 1-A. Multiple research studies^34,35^ have implemented multi-wavelength systems based on this principle of time-stretching technology in recent years, including radar signal matching filtering in the microwave photonics field^36–38^, perceptions^14^, and neural networks in the optical computing field^39–47^, etc.

However, the Kerr optical comb in the DCF-based system also possesses certain limitations. As shown in Fig. 1c, the uniformity of the comb teeth discussed in this paper is suboptimal, with a power disparity reaching 6.5 dB and 12.5 dB for 20 and 50 comb teeth, respectively. Previous studies have noted discrepancies between the actual computational outcomes in the system and theoretical predictions^41^. Despite employing a WS to initially equalize the intensity of various wavelengths, ensuring uniform input intensity into the system, discrepancies in the output results persist^14^. These discrepancies are also related to other factors. The DCF-based system involves multiple devices, including EDFA and DCF, which demonstrate distinct responses to varying wavelengths, leading to inconsistent attenuation effects across different wavelengths. Meanwhile, variations from the working environment are likely to influence the system responses as well, which is inevitable during the computing process.

To improve the accuracy of information processing in this system, it is essential to calibrate the intensity of the optical carriers at different wavelengths. There are several traditional methods such as dual-comb spectroscopy and spectrometers, used for the calibration and quantification of OFC. Each of these methods has its advantages and disadvantages. The equalization method using a spectrometer offers high resolution, making it suitable for combs with narrower repetition rates. However, its slow measurement speed hinders real-time monitoring. Dual-comb spectroscopy leverages the beat frequency effect of two combs to achieve high-resolution and high-speed spectral detection, suitable for rapid dynamic detection. However, it has high system complexity, requires strict phase synchronization, and becomes more complex to implement when the comb repetition rate is large.

This work presents the DRC method for the system, which enables calibration with minimal iterations, resulting in improved computational accuracy. Although the introduction of DCF increases attenuation, the structure is simpler compared to traditional methods. In this study, a WS modifies the signal intensities at different wavelengths, which is a key point to obtain good results using the DRC method. The modified signals are collected by a PD and measured by an oscilloscope. Simultaneously, system calibration is performed by the algorithm platform communicating wirelessly with both the oscilloscope and WS. The implementation method of DRC for the DCF-based system is presented in Supplementary Note 2-A.

Figure 1d displays the iterative cycles required for four calibration algorithms to achieve calibration under different situations of comb teeth. A 32-channel DFB laser array is also employed to generate different numbers of optical carriers, assessing the calibration speed of each algorithm within the system. The laser array features a wavelength spacing of 100 GHz. The 32 lasers cover a wavelength range from 1537.32 nm to 1562.92 nm. The power of each individual channel is around 0 dBm, and the line width is ≤3 MHz. By adjusting the attenuation at different wavelengths within the laser array, the inconsistency of an OFC is simulated. The data for PID and DRC are derived from 50 repeated experiments. Owing to the long iteration duration of the GA and SPGD, they underwent only 10 trials to gather the best iterative data.

Taking the case of 20 comb lines as an example, DRC achieves the target state in an average of 8.76 iterations, demonstrating the highest efficiency among the tested approaches. The PID method requires about 25.6 iterations to accomplish the same goal, ranking second in efficiency. However, both the GA and SPGD methods fail to calibrate the system within 300 iterations. As the number of combs increases, the average iterative cycle of each algorithm also shows an upward trend. The reason for this is that the increase in the number of combs leads to a further expansion of the spectral range, making the wavelength response inconsistency of the system more pronounced. This increases the difficulty of system calibration, resulting in a higher iteration cycle. However, even with a significant increase in the number of combs, the incremental increase in the iteration cycle for the DRC calibration algorithm remains small. This indicates that the calibration model trained in the simulated system can effectively reduce the number of iterations required during the calibration process.

Figures 1e, f compare the output before and after applying the DRC method, which clearly demonstrates a significant improvement of linearity post-correction. After calibration by DRC, the system errors are reduced by 78.0% and 44.6% of their initial value for 20 and 50 comb lines, respectively.

### Calibration of MRR-based System

**Fig. 2:**
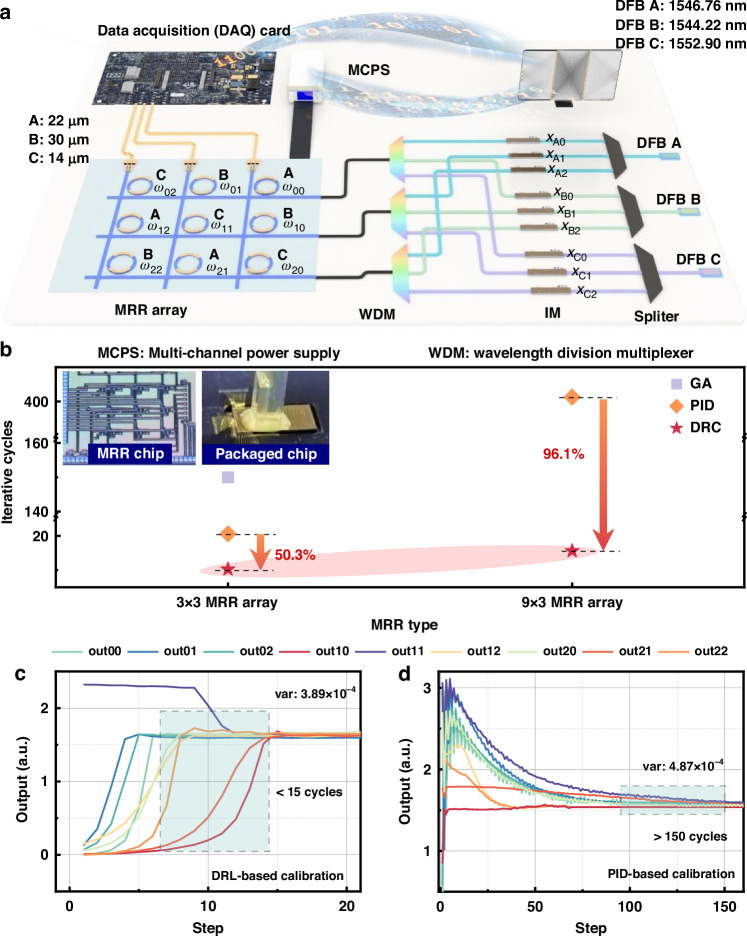
Calibration architecture and results for MRR array-based system. **a** shows the calibration link of the 3 × 3 MRR system. **b** illustrates the iterative cycles of various calibration algorithms applied across different MRR array systems. The top left insets present photographs of the 3 × 3 MRR array chip and the packaged chip. **c** and **d** demonstrate the outputs of different MRRs during the calibration process, utilizing DRC and PID calibration algorithms

Figure 2a presents the MRR array-based system, which is employed to validate the flexibility of the DRC algorithm proposed in this study further. The MRR array is designed by a 3 × 3 configuration, and the physical image is shown in the inset of Fig. 2b. The detailed information on the MRR chip design is shown in Supplementary Note 1-B. Recently, there has been significant advancement in the study of multi-wavelength systems using MRR technology^48–51^. The high integration and distinctive filtering capabilities of MRR have facilitated its widespread application in fields like microwave photonic filters^9,52^ and optical computing systems^53,54^.

Nonetheless, the theoretical and actual performance of MRR can differ because of design inaccuracies and manufacturing uncertainties. This discrepancy highlights the importance of precise calibration before deploying MRR arrays^55,56^. Without proper calibration, the actual output might significantly deviate from the expected one. This is the reason that calibration becomes even more critical in optical chips that integrate multiple MRRs, as inconsistent fabrication severely affects uniformity. In practical applications, it is usually expected for MRRs of identical diameter to show uniform wavelength and intensity responses. However, it is a challenge to meet this expectation in the real world, which has been proved by the large number of chip testing. Thus, to ensure smooth operation, calibration is necessary when deploying cascaded MRR chips in optical computing, where uniform response is crucial.

The execution of matrix convolution in this study is contingent upon the precise adjustment of the weights of MRRs. Prior to utilizing the MRR for modulation purposes, it is crucial to carefully examine the essential parameters. By employing a tunable laser, we methodically examine the wavelength response of 9 MRRs, with comprehensive scan results provided in Supplementary Note 3. Taking both cost-efficiency and system design into account, MRRs with identical diameters are paired with lasers processing the same wavelength. For each MRR, following the identification of the optical carrier, a multi-channel power supply (MCPS, TH-MS128-12CVFL) with a control precision of ±0.001 V is utilized to conduct voltage scans from 0 to 7 V, aiming to ascertain the effective voltage control spectrum. The application of a specific voltage induces a temperature elevation around the MRR, leading to a variation of its spectral response. This alteration is evidenced by the changed ratio of laser passing through the Drop and Through ports of the MRR, which is fundamental to the process of weight modulation in MRR. Based on the results of the voltage scanning, the voltage control range of each MRR can be determined. For each MRR, it is essential to confirm the location of the rightmost peak within the scanning voltage spectrum, which is designated as the start point for the voltage control range. The scanning process continues toward the right until the output nears zero, at which point it is established as the stop point of the voltage control range.

It is apparent that the peak values of voltage scanning and their respective voltage positions differ across various MRRs. Consequently, designing a specialized calibration algorithm is important to manage the response of different MRRs, and to reach a state of uniform weights efficiently. In essence, the primary objective is to identify the precise voltage values for each MRR that yield consistent power output through the Drop port. Implementing this calibration technique can significantly improve the accuracy of the chip in computational applications. The implementation method of DRC for the MRR array-based system is presented in Supplementary Note 2-B.

Figure 2b showcases the number of iterations required for calibration by four algorithms. The results are reliable as they are based on extensive testing, and each test involves hundreds of calibration trials. All algorithms utilize the same reward criteria to determine when the target state has been reached. Taking the 3 × 3 MRR array-based system as an example, the DRC approach averages 10.24 iterations to achieve calibration. The PID method necessitates an average of 20.6 iterations. Unfortunately, the GA and SPGD algorithms face significant challenges in attaining calibration for the MRR array-based system. The GA takes 150 iterations and the SPGD failis to do so even after 300 attempts. Thus, for the calibrations of the remaining two rows of MRRs, the less effective GA and SPGD methods are not considered to be viable options. To validate the scalability and effectiveness of the DRC algorithm on MRR array-based system, this method was deployed on a 9 × 3 MRR array. Figure 2b also presents the average number of iterations required for calibrating this chip using the DRC and PID methods. The iterations required for DRC and PID calibration are about 15.6 and 401.3, respectively. Detailed experimental information regarding the 9 × 3 MRR array can be found in the Supplementary Note 4.

For the 3 × 3 MRR array-based system, Fig. 2c, d depict the changes in the output states of each MRR during the calibration process of the three-row MRR array using the DRC and PID methods, respectively. It is evident that for the calibration of each row, the DRC algorithm reaches the target state in less than 10 rounds, whereas the PID approach requires at least twice as many iterations. This observation exhibits the efficiency and superiority of the DRC method again. Quantitative analysis reveals that the variances among the outputs of the system are 3.89 × 10^−4^ for the DRC method and 4.87 × 10^−4^ for the PID method. These results demonstrate the calibration performance of the four algorithms in the MRR array-based system. In terms of both speed and accuracy, the DRC approach outperforms the other methods.

### Calibration of MZI-based System

**Fig. 3:**
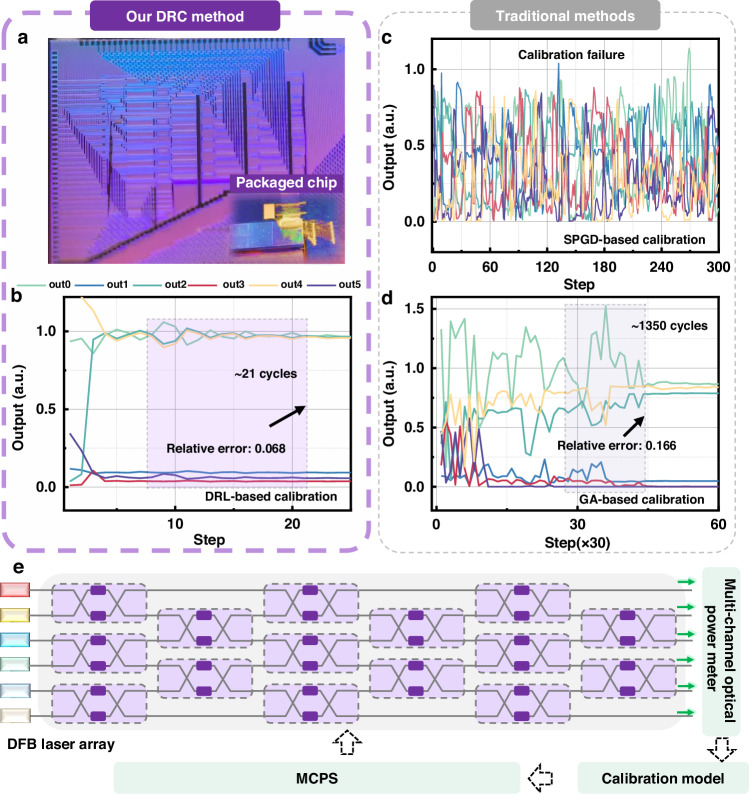
Calibration architecture and results for MZI array-based system. **a** presents a photograph of the MZI array chip. **b**–**d** demonstrate the outputs of the MZI array chip during the calibration process, utilizing DRC, GA and SPGD algorithms, respectively. **e** depicts the calibration link of the MZI array system

Figure 3e illustrates the MZI array-based system, which was similarly employed to validate the efficacy of the DRC calibration algorithm. This chip comprises 15 MZI units, each of which consists of two couplers and two phase shifters (PSs). The physical representation of the chip is shown in Fig. 3a. More design information about the MZI chip is provided in Supplementary Note 1-C. With the ongoing progress of optical computing technology, the application of MZI chips has expanded significantly^57–59^. They have been widely adopted in research efforts, contributing to the development of functional optical computing chips and their related applications^8,60,61^.

Prior to matrix encoding, it is common to scan the operating points of all MZI units to adjust the weights, but the processes are quite complex. In the context of multi-wavelength scenarios, the complexity of weight distribution is significantly increased. This work aims to calibrate the commonly used MZI array structures by employing the DRC algorithm. Unlike the previously mentioned systems, the parameters to be calibrated in this system do not have a one-to-one correspondence with the system output, complicating calibration. To effectively demonstrate the calibration results, the MZI array can be set to represent an identity matrix, establishing a one-to-one correspondence between input and output. The implementation method of the DRC algorithm is discussed in the Supplementary Note 2-C.

Figure 3b illustrates the changes in system output as the DRC algorithm is applied, with the input set to [1,0,1,0,1,0]. After approximately 21 iterations, the system rapidly achieved the desired target state, with a relative output error of 0.068, corresponding to an 85.4% reduction compared to the results obtained prior to calibration using the standard voltage configuration. This demonstrates the effectiveness of the DRC algorithm in calibrating the MZI array-based system.

The GA and SPGD calibration algorithms were implemented in the system, with the relevant results presented in Fig. 3c, d. The GA method requires approximately 1350 iterations for the system to reach a stable state, achieving a relative error of about 0.166. However, the SPGD method was unsuccessful in calibrating the system. In contrast, the DRC algorithm remains the superior method. It should be noted that MZI calibration is akin to solving underdetermined matrix problems, making PID unsuitable and thus not deployed.

### Impact of calibration errors on multi-wavelength system results

**Fig. 4:**
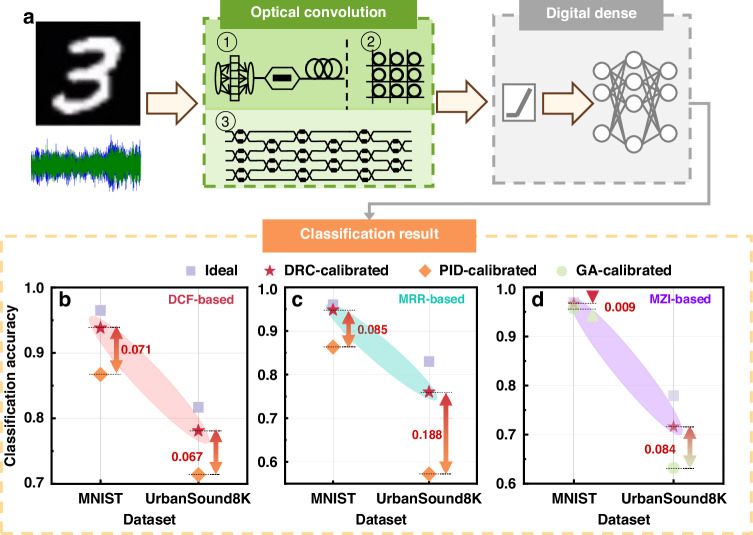
Comparison of classification accuracy of neural network models constructed based on different multi-wavelength systems under varying calibration errors. **a** demonstrates the architecture of the neural network based on different optical convolution units. **b**–**d** display the classification accuracy of neural network constructed with DCF-based, MRR-based and MZI-based systems after calibration with different algorithms, respectively

To assess the impact of several calibration methods on the accuracy of the three types of multi-wavelength systems in information processing, some simple neural network models were constructed using the corresponding operators of the system as the basis. As illustrated in Fig. 4a, a neural network framework combining a convolutional layer and a fully connected layer was constructed based on the aforementioned three types of multi-wavelength systems. These three distinct systems serve as convolutional kernels of varying sizes, enabling the implementation of optical convolution operations within the network model. Following the convolution process, the digital computer performs non-linear operations and the fully connected layer computations, ultimately facilitating classification tasks across different datasets. Upon completing the training of the neural network model, the average errors in different calibration algorithms were simulated as the weight errors of the convolutional kernel, resulting in different new neural network models. By comparing their performance in the same task, the effectiveness of the different calibration algorithms can be evaluated. Meanwhile, the ideal neural network is introduced, which is defined as a digital implementation of the same network architecture used in the calibrated photonic system, but realized entirely on a computer such that no numerical errors occur during training or inference. It serves as a baseline for evaluating how closely the performance of neural networks calibrated by different algorithms approaches that of an “ideal” system. The MNIST dataset^30^, which consists of grayscale images of handwritten digits in 10 categories, and the UrbanSound8K dataset^31^, which contains audio files in 10 categories typically found in urban settings, were chosen for classification tasks. Due to the differing nature of the tasks, the neural network models also varied accordingly. Subsequent paragraphs will elucidate the neural network models employed in each of the two systems.

For the DCF-based system, after dispersion in the long-distance DCF, multiple convolution kernels can be obtained by separating signals of different wavelengths using a wavelength division multiplexer (WDM)^39^. In the classification of the MNIST dataset, a WDM is designed to direct the first 10 optical combs and the last 10 optical combs to distinct detectors. Consequently, 2 convolution kernels are obtained, which can be construed as two 2 × 5 convolution kernels. When classifying the Urbansound8K dataset, the OFC can be split into four uniform groups using a WDM, and then each group is directed to a detector to obtain four 5 × 1 convolutional kernels. The Urbansound8K dataset is subjected to Mel-frequency cepstral coefficients (MFCC) transformation before being fed into the neural network for classification. As a commonly employed method for modeling audio signal features, MFCC finds applications across various domains^62^.

Experimental results show the classification accuracies of the neural network models obtained under different calibration algorithms in Fig. 4b. It is apparent that the best accuracy is achieved by the ideal neural network model for each dataset, with a minor decrease in accuracy for the DRC-calibrated model, and the lowest accuracy recorded for the PID-calibrated model. This indicates that the DRC method surpasses the PID approach in enhancing system accuracy.

To examine the impact of two calibration methods on the accuracy of the MRR array-based system in information processing, a neural network model was constructed using a 3 × 3 convolutional kernel operator as the foundation. Therefore, when designing the neural networks for classifying the MNIST and Urbansound8K datasets, it is sufficient to modify the size of each convolutional kernel to 3 × 3 while maintaining the rest of the network architecture. By repeatedly employing the MRR-based system, computations for multiple convolutional kernels within the network model can be realized. The comparative classification accuracy of the neural network models under different calibration algorithms is presented in Fig. 4c. The results indicate that the DRC method can be scaled to larger MRR arrays, and its calibration efficiency is significantly higher compared to the PID method.

For the MZI-based system, an arbitrary 6 × 6 weight matrix can be constructed through appropriate combinations, facilitating the execution of 6×6 convolution operations. Consequently, when the MZI array is employed as an optical convolution unit, the kernel size is set to 6 × 6. If multiple convolution kernels are required, this can be achieved by reusing the MZI array elements. Since the PID method is not applicable for the calibration of MZI arrays, this study compares the effectiveness of different calibration algorithms within an MZI system, specifically focusing on the GA algorithm and the DRC algorithm. The relevant results are illustrated in Fig. 4d. This result indicates that the network model calibrated using the DRC method exhibits significantly lower errors compared to the results obtained for GA calibration, resulting in classification accuracy that is closer to the ideal network model.

These outcomes suggest that a reduction in calibration error directly correlates with an increase in the accuracy of multi-wavelength optical information processing tasks. Previous experimental results have demonstrated that the DRC method exhibits a significant advantage in calibration accuracy compared to other traditional methods. Therefore, employing the DRC method for the calibration of multi-wavelength systems effectively enhances the reliability of optical information processing.

## Discussion

This study investigates four calibration methods within three multi-wavelength systems, categorizing them into heuristic and deterministic algorithms. Heuristic algorithms such as GA, SPGD, and DRC incorporate probabilistic approaches, making them suitable for tackling problems that are difficult to solve through conventional mathematical models or precise calculation methods, such as the calibration of MZI array-based systems. In contrast, deterministic algorithms like PID utilize precise strategies to attain optimal solutions for the calibration of DCF-based and MRR array-based systems. The experimental results indicate that the DRC method, which incorporates specific prior knowledge, demonstrates the highest calibration efficiency across all three types of multi-wavelength systems.

In evaluating the strengths and limitations, it is noted that the lack of prior knowledge in the GA extends its iteration time, while SPGD achieves faster convergence on certain problems but encounters difficulties with large-scale applications. PID showcases speed and simplicity in implementation, yet its effectiveness heavily relies on expert knowledge for parameter tuning. Conversely, DRC emerges as the superior method for real-time applications by rapidly achieving desired states through strategic policy selection, although it requires pre-training.

To conclude, this research focuses on utilizing a deep reinforcement learning algorithm to tackle the distortion caused by frequency-selective responses within multi-wavelength systems. The DRC approach significantly reduces output errors across various types of multi-wavelength systems, such as reducing output error in the DCF system to 22.0% of its original magnitude, decreasing output variance in the MRR array to 3.89 × 10^−4^, and minimizing relative output error in the MZI array to 14.6% of its manual calibration result. The DRC method produces effective calibration for all three systems by establishing a unified error model. The algorithm normalizes system outputs and control parameters. It also uses neural network-assisted parameter optimization. Experimental results show that the target calibration state is reached within 21 iterations. While special signals were employed during the calibration process, it is important to note that this algorithm is equally applicable to more complex and nonlinear input scenarios. For a more intricate discussion, the experimental and simulation details specifically for an MRR array-based system, can be found in the Supplementary Note 5. These comparative experimental findings underscore the superior calibration efficiency and accuracy of the DRC algorithm, suggesting its promising potential for enhancing calibration in microwave photonics and optical computing.

## Materials and methods

**Fig. 5:**
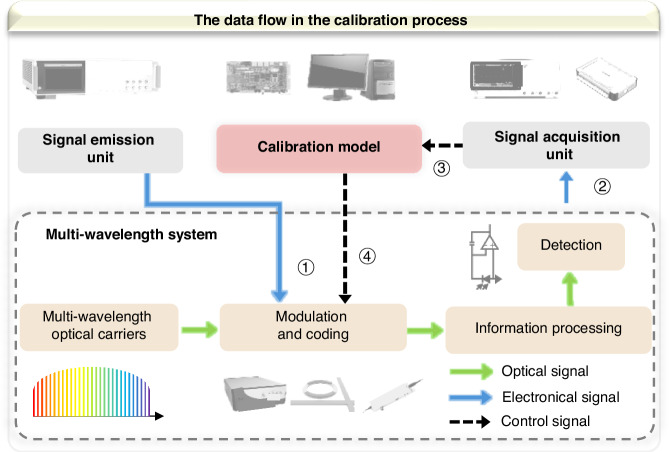
The flowchart of calibration processing in a multi-wavelength system. The yellow modules represent the components of the system, and the numbers denote the sequence of calibration steps

Figure 5 depicts the calibration processing model and multi-wavelength system developed in this study. The multi-wavelength system comprises four basic modules: multi-wavelength laser carriers, a modulation and coding unit, an information processing unit, and a detection unit. An OFC is used in the DCF system to provide numerous equally spaced carriers, aligning with the need for higher computational capacity. Conversely, the MRR and MZI systems demand stable output power and specific operating wavelengths, making DFB laser arrays more suitable. The calibration processing model mainly interacts with the modulation and coding unit, and the detection unit to exchange data. The calibration process initiates with feeding a standard signal into the modulation and coding unit of the multi-wavelength system via signal emission unit, aiming to build output signals that are easy to evaluate for errors. A signal acquisition unit captures the output signal from the multi-wavelength system, which is subsequently input into the calibration model. This model assesses the output signal for any irregularities. If the output does not match the expected result, the DRC algorithm is employed to compute calibration parameters. The DRC algorithm mainly calibrates components in the encoding unit, such as the driving voltage of the MRRs and MZIs, the input signal of the modulators, and the attenuation of different wavelengths in the WS. This method works on specific devices (including MZI, MRR, WS etc.) within the system to calibrate signals across various wavelengths. This calibration process is iterative and lasts until the output signal, which is obtained from the signal acquisition unit, reaches an acceptable level, indicating that the system has been calibrated successfully. A more detailed flowchart regarding the calibration process design can be found in the Supplementary Note 6.

The calibration model named DRC is designed and implemented with reference to the training strategies of DDPG^24^. The fundamental components of the DRC model include state, action, and reward. The state ${S}_{t}\in {{\mathbb{R}}}^{N}$ represents the normalized output state from the processed output of the multi-wavelength system, where $N$ denotes the number of output channels present in the system. Action ${A}_{t}\in {{\mathbb{R}}}^{M}$ corresponds to a change in the calibration parameters, with $M$ representing the number of calibration parameters being optimized. The reward function $R({S}_{t},{A}_{t})$ evaluates the alignment between the actual normalized output (${{\boldsymbol{y}}}_{{out}}$) and the ideal output (${{\boldsymbol{y}}}_{{ideal}}$), defined as:

1 $$R\left({S}_{t},{A}_{t}\right)=\alpha -\lambda \cdot {{\rm{||}}{{\boldsymbol{y}}}_{{out}}-{{\boldsymbol{y}}}_{{ideal}}{\rm{||}}}_{2}$$

where$\,{{||}\cdot {||}}_{2}$ denotes the L2 norm (Euclidean norm)^63^, $\alpha$ is the reward offset to set the baseline reward and $\lambda$ represents the scale factor for scaling the effect of the error term in the function. The use of the L2 norm is justified by its desirable mathematical properties: it is differentiable almost everywhere, encourages an overall minimization of error across all outputs in the system, and offers a stable gradient for optimization. For the hyperparameter $\alpha$, it is crucial to ensure that the reward remains non-negative when the system reaches the desired state, thereby accelerating convergence. Additionally, careful tuning of the hyperparameter $\lambda$ is necessary to enhance sensitivity to errors between the output and the ideal, ensuring effective reduction of deviations. The values of these two parameters should be configured based on the specifics of the system and empirical observations. Detailed definitions and discussions on the reward formulations for the DCF, MRR, and MZI systems are provided in Supplementary Note 2.

The DRC model utilizes a dual-network framework comprising the actor network and the target actor network, both implemented as four-layer fully connected neural networks. The actor network takes the environmental state information as input and generates an action strategy ${A}_{t}={Actor}({S}_{t})$ that aligns with the objective. The target actor network is specifically used to train and optimize the parameters of the calibrating policy. During the calibration process, the parameters will also be iteratively updated. A replay buffer is used to store the system inputs and outputs obtained during the actual calibration process, containing $({S}_{t},{A}_{t},R\left({S}_{t},{A}_{t}\right),{S}_{t+1})$. These data will serve as the training dataset to further optimize the actor model. Iterative updates of the target actor network enable the DRC model to converge toward an effective calibration strategy that minimizes error across various optical systems, thereby enhancing overall system stability and performance. More details about the interference and training phase of the DRC model are shown in Supplementary Note 6.

It is worth noting that the three algorithms used for horizontal performance comparison, namely GA, SPGD, and PID, are all based on uniform hardware. Detailed information regarding the design and implementation of the three algorithms can be found in Supplementary Note 7.

## Supplementary information

Supplementary Information for Multi-Wavelength Optical Information Processing with Deep Reinforcement Learning

## Data Availability

The data that support the findings of this study are available from the corresponding authors upon request.

## Code availability

The codes that support the findings of this study are available from the corresponding authors upon request.
