# Supplementary material for: Multi-wavelength optical information processing with deep reinforcement learning
Source: Light Sci Appl. 2025 Apr 15;14:160. doi: 10.1038/s41377-025-01846-6 (PMC11997129; doi:10.1038/s41377-025-01846-6)
Supplement: Supplementary file 1 — Supplementary Information for Multi-Wavelength Optical Information Processing with Deep Reinforcement Learning [file 41377_2025_1846_MOESM1_ESM.pdf]

Supplementary Information for Multi-Wavelength Optical Information Processing with Deep Reinforcement Learning

Qiuquan Yan[1,†], Hao Ouyang[2,†], Zilong Tao[1], Meili Shen[3], Shiyin Du[1], Jun Zhang[3,], Hengzhu Liu[1], Hao Hao[2,*] and Tian Jiang[2,4,5,*]*
[1]College of Computer Science and Technology, National University of Defense Technology, Changsha 410073, China
[2]Institute for Quantum Science and Technology, College of Science, National University of Defense Technology, Changsha 410073, China
[3]National Innovation Institute of Defense Technology, Academy of Military Science PLA, Beijing 100071, China
[4]College of Advanced Interdisciplinary Studies, National University of Defense Technology, Changsha 410073, China
[5]Hunan Research Center of the Basic Discipline for Physical States, National University of Defense Technology, Changsha 410073, China
Correspondence: Professor Jun Zhang, E-mail: zhangjun12a@nudt.edu.cn
Correspondence: Professor Hao Hao, E-mail: HH65637917@163.com
Correspondence: Professor Tian Jiang, Email: tjiang@nudt.edu.cn
[†]Those authors contributed equally to this work.

The Supplementary includes:

Supplementary Note 1. Structure details of three types of multi-wavelength systems.

Supplementary Note 2. Implementation methods of the task-specific DRC algorithm.

Supplementary Note 3. Microring characterization.

Supplementary Note 4. Calibration of the 9×3 MRR array-based system.

Supplementary Note 5. Effectiveness of the DRC algorithm in complex signals.

Supplementary Note 6. Calibration process details and calibration models.

Supplementary Note 7. Fundamentals of calibration methods based on PID, GA, and SPGD algorithms.

1. STRUCTURE DETAILS OF THREE TYPES OF MULTI-WAVELENGTH SYSTEMS

A. DCF-based System

In the OFC of the DFC-based system, a continuous wave pump source at 1550.12 nm is produced by a distributed feedback (DFB) laser and amplified to 25.8 dBm through an Erbium-Doped Fiber Amplifier (EDFA). The optical signal enters an MRR, undergoing resonant amplification to kilowatt levels. The Kerr nonlinearity triggers both degenerate and non-degenerate four-wave mixing, leading to the spontaneous creation of equidistant multi-level sidebands and a frequency comb with a distinct multi-toothed appearance. The MRR, composed of Si_3N_4, features a free spectral range (FSR) of 100.3 GHz and a Q-factor of approximately 3×10^6. The OFC passes through a fiber Bragg grating (FBG) to filter out the strong pump signal, producing a single-soliton optical comb spectrum.

The system employs the OFC as the multi-wavelength laser source and utilizes a WSS to modulate the light intensity, thereby preprocessing the optical comb. The WSS operates in the C-band with a minimum filter bandwidth of 10 GHz, making it suitable for configuring weights for the OFC with a 100 GHz repetition rate interval. Its attenuation range is 0-35 dB with a resolution of 0.01 dB. The modulated signal, denoted as W ($W = [\omega_0, \omega_1, \cdots, \omega_{N-1}]$), undergoes further processing. Subsequently, different types of information X ($X = [x_0, x_1, x_2, \cdots]$) intended for processing are modulated onto each optical carrier as electrical signals through an intensity modulator. The modulator is connected to a long-distance DCF, which introduces a consistent time delay $\Delta\tau$ between adjacent wavelengths. The calculation of $\Delta\tau$ can be performed as $\Delta\tau \approx D \times \Delta n \times l$, where D symbolizes the dispersion coefficient of the DCF, Δn denotes the spacing between adjacent wavelengths, and l represents the length of the DCF.

In 1997, Tong et al. designed a method for measuring fiber dispersion[1], followed by Jalali et al., who utilized DCF to achieve spectral measurement in the time domain[2]. This also marked the inception of time-stretch technology based on DCF. For ease of result presentation, this study employs a DCF with a dispersion coefficient near -133 ps $(\mathrm{km} \cdot \mathrm{nm})^{-1}$ and a length approximately 9.203 km. Given the OFC spacing of around 0.8 nm, the time delay for adjacent optical comb arrivals at the photodetector (PD) is roughly 1 ns. With a modulator signal input rate of 1 GHz, the delay between the adjacent optical comb arriving at the PD end is exactly one symbol. The time-stretching technology forms the basis for optical information processing in this system. For instance, with N optical combs, the calculation process performed by this system is shown as $S = W * X$, where $s_t = \sum_{i=0}^{N-1} \omega_i x_{i+t}$. The symbol $*$ denotes the vector convolution operation, and s_t represents the calculation result at time t.

B. MRR aray-based System

In the MRR array-based system, the total footprint of the chip used in the study is 7.9×4.3 mm², with the dimensions of MRR chip being approximately 1.2×1.2 mm². The chip fabrication process utilizes a 180 nm process technology node on a 220 nm standard silicon-on-insulator platform. The MRR chip mentioned in this study was custom-designed by us and fabricated on an 8-inch wafer with a thickness of 220 nm. The waveguide width of the MRR is 450 nm, and the gap between the ring waveguide and the straight waveguide is designed to be 250 nm to ensure the MRR is as close to critically coupled as possible. To

prevent interference between the simultaneously processed wavelengths, the diameters of the MRRs were set to 14, 22, and 30 ¯m, respectively. Furthermore, to introduce diverse diameters across columns, the configuration of each row in MRRs is distinct. This design permits the output end of each column to process three different wavelengths at the same time, thereby significantly increasing the processing capabilities of the chip. To ensure the stable operation of the MRR array, a temperature electronic controller is used to maintain the overall chip temperature at 25 °C. The laser source is comprised of three DFB lasers, providing each row of MRRs with input from three intensity-modulated optical carriers. At the Drop end of each column, PD and TransImpedance Amplifiers (TIA) are integrated to enhance the sensitivity of optical signal detection. Subsequently, the outputs are captured by a data acquisition card. By aggregating the output intensities from three columns, the convolution result of the matrix is obtained. This type of operation is prevalent in convolutional neural networks (CNNs) that are used for image processing. The MRR array, organized in a 3×3 configuration, effectively mimics a 3×3 convolution kernel. This configuration allows the chip to perform convolution operations on two-dimensional matrices, making it a valuable tool for various image processing applications.

C. MZI array-based System

For the MZI array-based system, the total area of the MZI chip is approximately 5×10 mm^2, with the MZI array occupying about 5.64 mm^2. The chip was designed and fabricated using a 180 nm process on a standard 8-inch silicon-on-insulator platform. It supports six inputs of optical signals and six outputs of optical signals, allowing the theoretical construction of any 6×6 unitary matrix. Its efficient encoding capabilities in matrix operations have led to its utilization in investigations of optical computing architectures.

2. IMPLEMENTATION METHODS OF THE TASK-SPECIFIC DRC ALGORITHM

A. DCF-based System

To implement the DRC method effectively, it is crucial to specify the input and output dimensions of the system in accordance with the action and state dimensions of the policy network. In the DCF-based system, the input dimension of the system is determined by the number of comb teeth. To test DRC method, 20 comb teeth are selected here which are shown in Fig. S1. Thus, the input parameter $P = [attn_0, attn_1, \cdots, attn_{19}]$, with $attn_i$ indicating the attenuation for the ith optical comb tooth. The output dimension is formed by reference output of the system. Ideally, 20 modulated square waves of optical carriers are generated which are shown in Fig. S2a, when the uniform intensities of OFC (i.e., $W = [1, 1, \cdots, 1]$) are achieved and a square-wave signal of 40 ns period is input using an IM. After passing through a fixed time delay, a delay of one symbol is formed between the adjacent optical combs, as depicted in Fig. S2b-c shows the theoretical calculation result, highlighting 20 rising edges within the periodic output signal. Each step maintains the same height difference. Given that the output of system and each cycle output is periodic and symmetrical, the accuracy of the calculated result examined only by the rising edges is feasible. The output parameter $output = [out_0, out_1, \cdots, out_{20}]$, where out_0 signifies the minimum output of the cycle, and out_{20} represents the maximum output.

To accurately simulate this system and generate a synthetic dataset, it is crucial

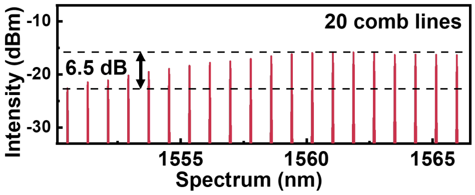

Fig. S1. The 20 optical comb lines used in the system which were generated from the Kerr optical comb.

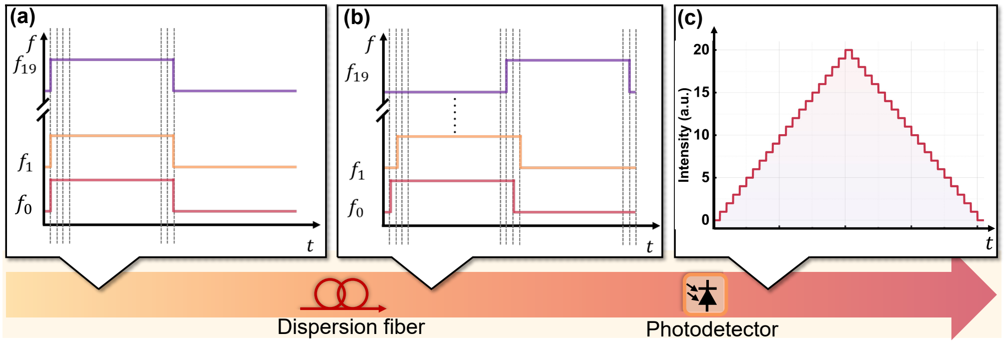

Fig. S2. Ideal processing results for DCF-based system. (a) illustrates a square wave signal modulating 20 optical combs concurrently. (b) delineates the delay of one symbol among all adjacent comb teeth subsequent to traversing the dispersion fiber. (c) elucidates the theoretical calculation outcomes of one cycle output by the PD.

to construct appropriate transfer functions between the normalized output data and the policy action. The action vector is represented by $A = [a_0, a_1, \cdots, a_{19}]$, with a specified mapping relationship between A and P_{update} written as follows:

$$P_{update} = (P + \lambda \cdot A) - min(P + \lambda \cdot A) \tag{S1}$$

where λ can be fine-tuned based on the specific conditions of the system, *parameter* means the last result. Given that the output variation between consecutive steps directly matches the relative strengths of the OFC, the state vector is formulated as $S = [s_0, s_1, \cdots, s_{19}]$, with the following relationship to the *output*:

$$\begin{aligned} s_i &= out_{i+1} - out_i \; (i = 0, 1, \cdots, 19) \\ S &= \frac{S}{\min(S)} - 1 \end{aligned} \tag{S2}$$

S represents the proportion of the intensities of the optical combs that exceeds the minimum value. The action A similarly shows a nearly positive relationship to the power reduction needed for all wavelength in the present state. The policy network, known as the actor model, aims to closely estimate a model that takes the S as input and generates the A as output. The actor model utilizes a four-layer fully connected neural network built with TensorFlow. It features an input layer that receives a state vector of length 20 and an output layer that delivers an action vector of the same dimension. For training purposes, a dataset that hypothesizes a linear correlation between S and A values is compiled due to their similarities. A certain level of randomness to simulate random noise is

introduced to the A in the environment. Moreover, the range of state is defined as $[0, 8]$, considering the diverse power levels exhibited by the optical combs in practice. Within this range, 2000 value pairs are randomly produced for training, and 500 pairs in which for testing. During the training phase of target actor model, the batch size is fixed at 32, with 200 training epochs, employing Adam as the optimizer and Root Mean Squared Error (RMSE) as the loss function:

$$loss = \sqrt{\frac{\sum_{i=0}^{n-1} (y_i - \hat{y}_i)^2}{n}} \tag{S3}$$

where y_i denotes the predicted value and $\hat{y}_i$ signifies the actual result.

The variation curve of the loss value in target actor model depicted in Fig. S3a demonstrates a good alignment with the training goals. The enlarged diagram shows that the network can achieve the training objective with minimal overhead. Despite some deviations between the output of target actor model and theoretical expectations, this error can serve as a stochastic approach. It resembles the ϵ-greedy approach used in reinforcement learning training to enhance policy optimization.

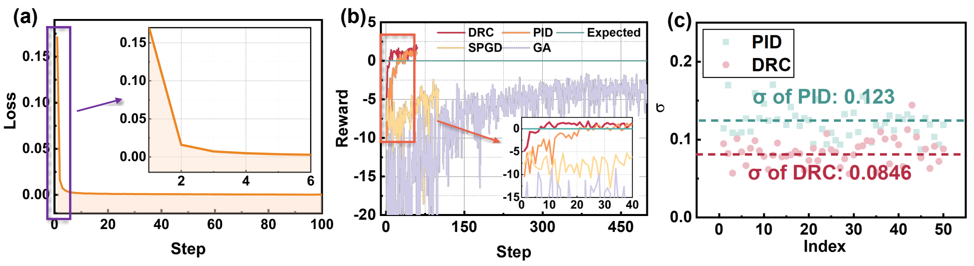

Fig. S3. Calibration processing results for DCF-based system. (a) exhibits the alteration in loss throughout the training of the strategy network. (b) outlines the reward variations during the iteration process predicated on 4 disparate algorithms for calibration methods. The modifications in *reward* for the initial 40 rounds are enhanced and presented. (c) presents a comparative analysis of system errors between the PID and DRC algorithms following 50 repeated calibration experiments.

Once the training of the target actor model is completed, its parameters are fully copied to the actor model. The actor model is then deployed in the system to execute the DRC algorithm. The reward function of the system for guiding the DRC process is characterized as follows:

$$reward = 3 - 15 \times \sqrt{\frac{\sum_{i=0}^{19} (s_i - \bar{s})^2}{20}} \tag{S4}$$

$\bar{s}$ denotes the mean value of the S. A higher *reward* implies a more regular intensity change between consecutive steps in the output of system, enhancing the wavelength uniformity. A *reward* greater than 0 indicates the system has reached its desired state. This reward function is utilized to evaluate the effectiveness of four distinct algorithms in achieving the target state through calibration. The parameter 3 and 15 were added to the reward function to meet this requirement. Figure S3b illustrates the variations in reward during the

operation of diverse calibration methodologies. From the enlarged illustrations, significant fluctuations are observed in *reward* enhancement in other algorithms.

Figure S3c presents a comparative analysis of output errors from 50 repeated experiments utilizing calibration algorithms derived from PID and DRC methodologies.To evaluate the calibration output errors in the DCF-based systems, let $\tilde{\mathbf{y}}_{out}$ represent the actual normalized output vector of length N. The $\tilde{\mathbf{y}}_{target}$ means the normalized target output vector. The relative differences e_i are expressed as: $e_i = \frac{\tilde{y}_{out_{i+1}} - \tilde{y}_{out_i}}{\tilde{y}_{target_{i+1}} - \tilde{y}_{target_i}}$ for $i = 0, 1, \cdots, N-1$. The mean of those relative difference is given by: $\mu = \frac{1}{N}\sum_{i=0}^{N-1} e_i$. The overall error is then calculated using the standard deviation: $Error_{DCF} = \sqrt{\frac{1}{N}\sum_{i=0}^{N-1}(e_i - \mu)^2}$. The average error rates in system outputs after calibration by these two approaches are recorded at 0.123 and 0.0846, respectively.

During the calibration of the DCF-based system, if there is a change in number of comb teeth of the multi-wavelength optical source, only the sizes of the *actor* and *state* need to be adjusted to correspond with the new number of comb teeth. The remaining processing steps remain consistent with those previously described.

B. MRR array-based System

According to a similar concept with DCF-based system, this study also developed the DRC method for chip calibration. During the calibration phase, the signal emission unit is connected to the RF input of the intensity modulator within the system. The standard signal input is set to 1. This standard input is benefit to evaluate the output consistency of system. Signal acquisition is performed using an Smacq 5121 data acquisition card at a 500 kSa/s sampling rate. The data processing unit collects output electrical signals from the three Drop ports of the chip, to access the conformity of outputs from different MRRs with the targeted value. The voltage of MRRs is adjusted through an MCPS to modulate the corresponding weights. However, simultaneously controlling nine MRRs for calibration is demanding, as the signal from each row results from three modulated MRRs. To address this problem, this work adopts a sequential strategy, calibrating MRRs within the same row individually, and scanning each row to calibrate all nine MRRs. To improve the accuracy of system, it is essential to maximize the output signals from all MRRs. By comparing the peak values of each MRR, it was observed that the MRR in the second row exhibits the lowest peak. Consequently, given an identical optical power input, the expected value of all MRR outputs is set to the lowest peak value in the second row. Therefore, the target state of the second row is selected to be the minimum peak state, while the calibration states for the other two rows of MRRs are non-maximum state.

Next, we outline the parameters employed in the DRC model. The system input, denoted as $V = [v_0, v_1, v_2]$, symbolizes the relative voltages across three MRRs within the same row. The relationship between the output of the policy network, $A = [a_0, a_1, a_2]$, and V is related to the calibration control of the MRRs. During the calibration phase for the second-row MRRs, identifying their minimum peak is necessary. The relationship between these components is

explained below:

$$\begin{aligned} \theta =& \frac{\min(A+1)}{\max(A+1)} \\ V =& V + \theta \cdot A \\ V =& V - \min(V) \end{aligned} \tag{S5}$$

During the calibration of the remaining two rows of MRR, their interrelation is as follows:

$$V = V - \beta \cdot (\min(v_{max}) - A) \tag{S6}$$

$\min(v_{max})$ represents the lowest peak value obtained from the second row. After calculating the relative voltage values, these values are modified to align with the adjustable range of each MRR and then set accordingly for each MRR. The system generates three output voltages, represented as $output = [out_0, out_1, out_2]$, which directly serve as the input, state $S = output$, for the policy network. Similar to the previous section, there exists a resemblance between S and A. Building the simulation dataset in an identical manner, is utilized for training the policy network. The parameters for network training are similar with those outlined in the previous subsection, expect that the input and output dimensions of the policy network are different in the MRR array-based system.

Once the policy model training is completed, it is deployed on the MRR array for calibration purposes. The reward function designed for the MRR calibration, specifically in the second row, is defined as follows:

$$reward = \bar{s} - 50 \times \sqrt{\frac{\sum_{i=0}^{2}(s_i - \bar{s})^2}{3}} \tag{S7}$$

The greater the reward, the more consistent are observed among the triple outcomes. When the reward surpasses 0, the desired state is attained. The parameter 50 is to ensure that when the system output error is within the expected error range, it satisfies $reward \geq 0$. The smaller the error, the greater the $reward$. Figure S4a illustrates the changes in reward during iteration for the four calibration methods employed in the MRR array-based system. Figure S4b shows the system output variance for the PID and DRC methods, repeated across 50 experiments. To assess the error in the MRR-based system, the ratio between the actual and target outputs for each channel is considered. First, define $\mu = \frac{1}{N}\sum_{i=0}^{N-1}(\frac{\tilde{y}_{out_i}}{\tilde{y}_{target_i}})$ as the mean of the system outputs. The error $Error_{MRR}$ is computed as the total variance: $Error_{MRR} = \frac{1}{N}\sum_{i=0}^{N-1}(\frac{\tilde{y}_{out_i}}{\tilde{y}_{target_i}} - \mu)^2$. The average standard deviations obtained by the two methods are about 0.026 and 0.009, respectively. This comparison further validates the superior correction accuracy of DRC than the PID approach.

To calibrate the remaining two rows of MRRs, the modulation needs to be adjusted to a non-maximum output state. Consequently, the evaluation function should be modified to adapt to this change. The updated reward function is defined as follows:

$$reward = 0.1 - |\bar{s} - \min(v_{max})| - 50 \times \sqrt{\frac{\sum_{i=0}^{2}(s_i - \bar{s})^2}{3}} \tag{S8}$$

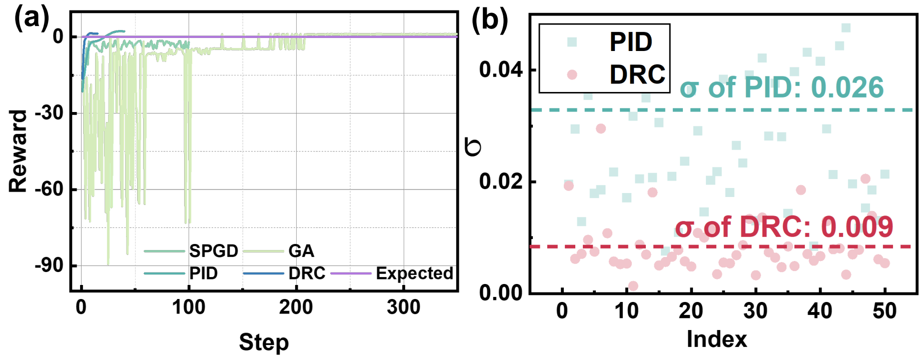

Fig. S4. Calibration processing results for MRR array-based system. (a) shows the change in reward during the iteration process based on four distinct calibration methods. (b) provides a comparative analysis of the output variance between the PID and DRC methods following 50 repeated calibration experiments.

By enlarging the reward, the outputs of the three MRRs are closer to the desired value $\min(v_{max})$. This leads to increased uniformity among the MRRs. Experimental results show that when the reward is greater than 0, the system output basically reaches the desired state. It also indicates that the each row has been successfully corrected. This is because the deviation between the output signals and the desired value has been minimized to an acceptable level.

C. MZI array-based System

To complete the calibration of the MZI array-based system, it is essential to first determine the operating point of each phase shifter (PS) on the chip. This work employs a programmed automated voltage scanning method to calibrate the operating points of the PSs within the MZI array. Similar to the configuration of the MRR, the voltage operating range for each PS is defined by the voltage ranges of the adjacent cross and bar states along the PS's scan curve. Although each MZI unit consists of two PSs, only one PS per MZI unit is controlled during operation to adjust its state. Therefore, while calibrating the MZI array system, the output of the system is modified solely by adjusting the operating voltages of the 15 PSs across the MZI array through a calibration algorithm.

During the calibration process, a 6-channel switch-controlled DFB laser array serves as the laser source for the MZI system, with the wavelengths of the six lasers being spaced at 1.6 nm apart, starting from a wavelength of 1549.32 nm. A multi-channel optical power meter (MCOPM) is utilized to detect the output signal power from the six channels of the system. A computer remotely controls the MCOPM to obtain output results, executing the DRC algorithm to determine the voltage calibration values for the PSs, which are then fed back into the MCPS to adjust the voltage values of the 15 PSs. The DRC algorithm is executed iteratively until the output results reach the desired state, thus completing the system calibration.

In the DRC algorithm, the input ($V = [v_0, v_1, \cdots, v_{14}]$) to the MZI array chip corresponds to the operating voltages of the PSs on each arm of the MZI array. The length of the output action generated by the policy network is also 15, and the results are mapped to the operating voltage range of each PS, producing the corresponding values in V. The output of the MZI chip is

$Out = [out_0, out_1, \cdots, out_5]$, and the relationship with the input state (S) of the policy network is given by: $S = Attn - Output - In_{power}$, where In_{power} represents the six inputs to the chip, and $Attn$ denotes the attenuation power on the chip. Since there is no one-to-one correspondence between S and A at this point, there is no linear relationship. During the training stage, designing a simulated dataset is quite challenging. Thus, for the DRC algorithm, the training mode of the policy network completely relies on the actual sampled data in the link. The training strategy follows the DDPG model, utilizing two critic networks and two actor networks to collaboratively train the policy model within the actual system. The two critic networks share the same architecture, both consisting of four layers of fully connected neural networks, with an input vector dimension of 20 and an output dimension of 1. The loss function for the critic network is defined as follows:

$$Loss_c = \frac{1}{n}\sum_{i=1}^{n}(Q_{predict\_i} - (r_i + \gamma Q_{target\_i})) \tag{S9}$$

Here, n represents the batch size, $Q_{predict\_i}$ refers to the Q-value obtained from the critic network, r_i denotes the reward for the i-th action, and Q_{target_i} is the Q-value acquired from the target critic network.

Similarly, the two actor networks have an identical structure, also composed of four layers of fully connected neural networks, with an input vector dimension of 6 and an output dimension of 15. The loss function for the actor network is defined as:

$$Loss_a = -\frac{1}{n}\sum_{i=1}^{n} critic(s_i, a_i) \tag{S10}$$

This function represents the negative sum of Q-values calculated from critic network for all actions. Each of the four networks contains hidden layers that consist of 128, 256, and 128 neurons, respectively.

For the MZI array, the reward function during the calibration process is defined as $reward = 0.01 - \sum_{i=0}^{N-1} |s_i|$. Theoretically, as the value of S approaches 0, the system converges towards the desired state. Consequently, a reward value that approaches 0 indicates an improved calibration of the system state. In this work, the threshold for the reward is set at 0, signifying that the system calibration is considered complete when the cumulative power error across six channels does not exceed 0.01.

The error in the MZI system is obtained by summing the absolute differences over all channels: $Error_{MZI} = \sum_{i=0}^{N-1} |\tilde{y}_{out_i} - \tilde{y}_{target_i}|$. Given that the relative values of the outputs can vary under different calibration methods, this relative output error is employed to assess the system's output performance.

3. MICRORING CHARACTERIZATION

To determine the operating wavelengths of individual MRRs in the MRR array, a wavelength scan must be conducted for each MRR initially. This process involves using a tunable laser to scan every MRR while monitoring the laser intensity output at the Drop port. The input laser power is maintained at 0 dBm with the scanning spectrum extending from 1525 nm to 1565 nm. The outcomes of the scans are depicted in Fig. S5. To optimize the use of optical carriers, it is advantageous to select MRRs with identical diameters that operate at similar wavelengths. According to Fig. S5, MRRs with diameters of 14 nm, 22 nm, and

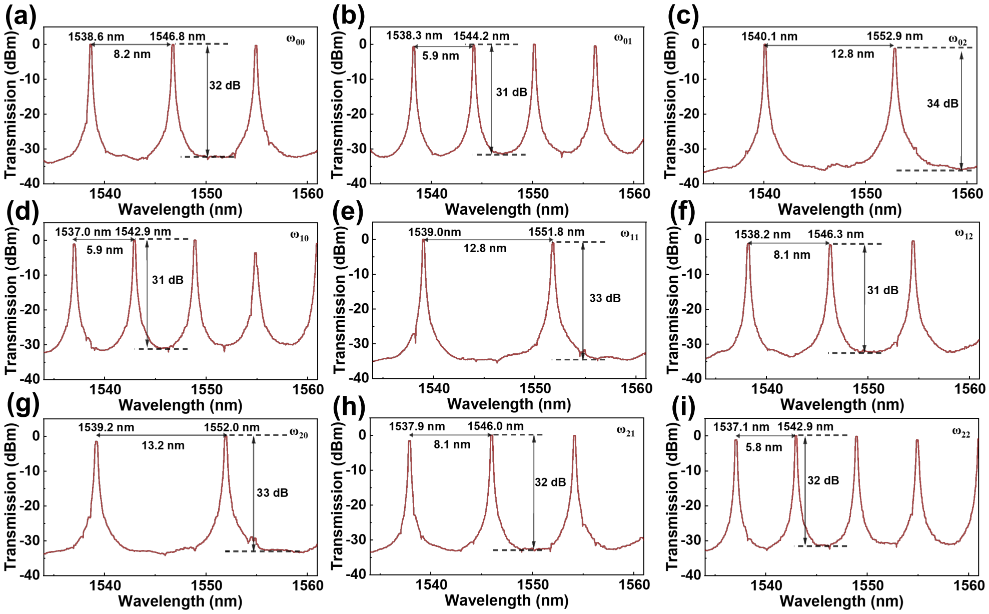

Fig. S5. Transmission curves of all MRRs in the chip.

30 nm are chosen to operate at wavelengths of 1552.90 nm, 1546.76 nm, and 1544.22 nm, respectively. This necessitates a significant voltage offset for certain MRRs, leading to increased power consumption.

It is worth mentioning that the detailed theoretical results $f(W, X)$ for the outcomes at the three output ports in the 3 × 3 MRR chip are provided as follows:

$$f(W,X) = \begin{bmatrix} \omega_{00} \cdot x_{A0} & \omega_{01} \cdot x_{B0} & \omega_{02} \cdot x_{C0} \\ + & + & + \\ \omega_{10} \cdot x_{B1} & \omega_{11} \cdot x_{C1} & \omega_{12} \cdot x_{A1} \\ + & + & + \\ \omega_{20} \cdot x_{C2} & \omega_{21} \cdot x_{A2} & \omega_{22} \cdot x_{B2} \end{bmatrix} \tag{S11}$$

where W denotes the modulation weights applied to different MRRs, X symbolizes the signal intensity adjusted by the intensity modulator, while $f(W, X)$ means the computing result.

4. CALIBRATION OF THE 9×3 MRR ARRAY-BASED SYSTEM

The structure of the 9×3 MRR array is shown in Fig. S6. The fabrication process of the chip is consistent with that of the 3×3 MRR array, and the core area is approximately 3.6×1.2 mm². To ensure that different MRRs operate at different wavelengths, the diameters of the MRRs in the same row are designed to be different. In this chip, the diameters of the MRRs in the first row are 16, 18, 20, 22, 24, 26, 28, 30, and 14 μm, respectively. MRRs with the same diameter are illustrated in the same color. The MRRs in different rows are arranged in a cyclic shifted manner to ensure that the MRRs in the same column have different

diameters. The left-side channels can input 9 different wavelength optical carriers, each corresponding to the operating wavelength of MRRs of different colors. Each column of MRRs on the chip integrates a PD and transimpedance amplifier.

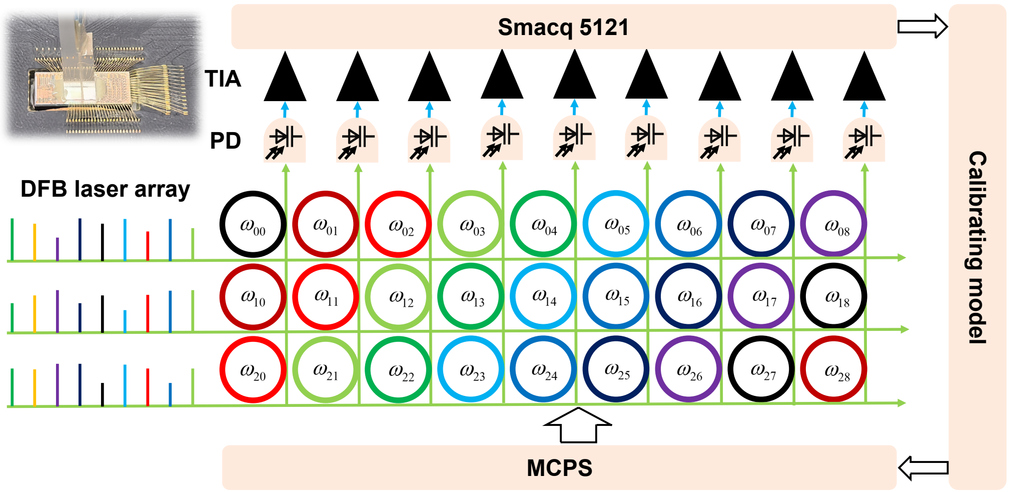

Fig. S6. Calibration structure diagram of the 9×3 MRR array-based system. The inset in the upper left corner is a photograph of the chip after packaging.

Here introduce the calibration process of the chip using the DRC method. Firstly, a tunable laser was used to scan the all MRRs to differentiate their operating wavelengths. Ultimately, wavelengths ranging from 1549.32 nm to 1562.13 nm with 200 GHz intervals were selected as the operating wavelengths and appropriately adjusting the weights of all the MRRs, a convolution operation on a 9×3 scale can be realized. Subsequently, the voltage of each MRR was scanned individually to determine its operating range. Upon the completion of scanning, the row with the minimum peak value of the MRRs, which corresponds to the second row of this chip, can also be identified. Thereafter, a calibration model was constructed and a simulated dataset was generated for pre-training.

Figure S7 illustrates the changes in MRR outputs during the execution of the calibration process based on DRC and PID algorithms. Figure S7a-c demonstrate the output variations of different MRRs during the DRC calibration, while Fig. S7d-f depict the output variations during the PID calibration process. Quantitative calculations reveal that the system output variances after calibration using two algorithms are approximately 1.33×10^{-5} and 7.30×10^{-5}, respectively. Compared to the 3×3 MRR array, the calibration of the 9×3 MRR array is evidently more challenging. The numebr of iterations for PID calibration has increased by an order of magnitude. The advantage of the DRC is more apparent. Although the size of the chip is three times larger than before, the number of calibration iterations required is only 1.5 times more than before. This also reflects the superiority of the DRC algorithm.

5. EFFECTIVENESS OF THE DRC ALGORITHM IN COMPLEX SIGNALS

A. Calibration under Linear Response Conditions

In a system operating under linear response conditions, calibration based on a set of standard inputs can be applied to other arbitrary inputs. This phenomenon

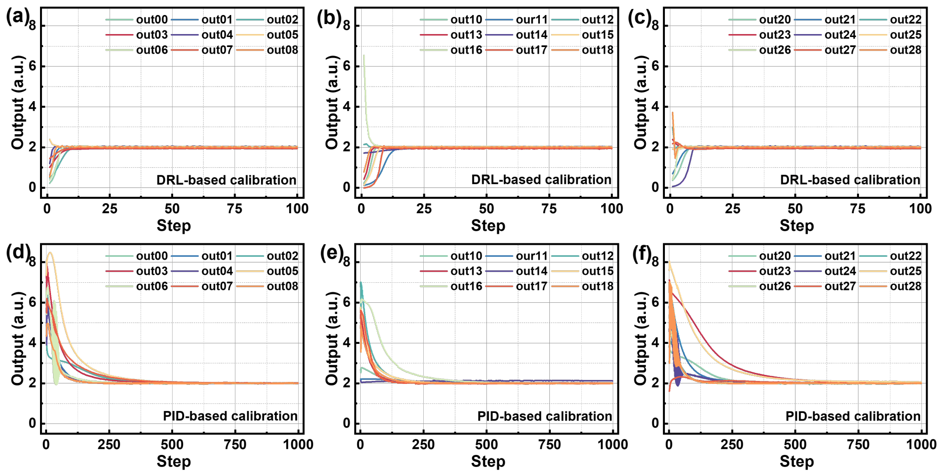

Fig. S7. Changes in MRR outputs during the calibration iterations. (a)-(c) show the MRR outputs during the DRC calibration process, and (d)-(f) show the MRR outputs during the PID calibration process.

arises from the closure properties of linear response systems with respect to addition and scalar multiplication. Therefore, after calibration with the standard input X_s, the system response $F_{chip}(X, W)$ can be expanded to accommodate any input.

For the system based on a 3×3 micro-ring, after calibration, the inputs X are randomly generated and mapped to the optical power across different micro-rings. The experimental data which are shown in Figure S8 collected from the accumulated calculations along three channels are compared with theoretical results. A total of 100 random input sets are generated, and the variances of the errors between the theoretical values and the actual outputs are calculated, yielding values of 1.02×10^{-3}, 9.9×10^{-4} and 1.01×10^{-3} for the three channels, respectively. Within a certain error range, the system response to any complex input remains fundamentally consistent with the theoretical results.

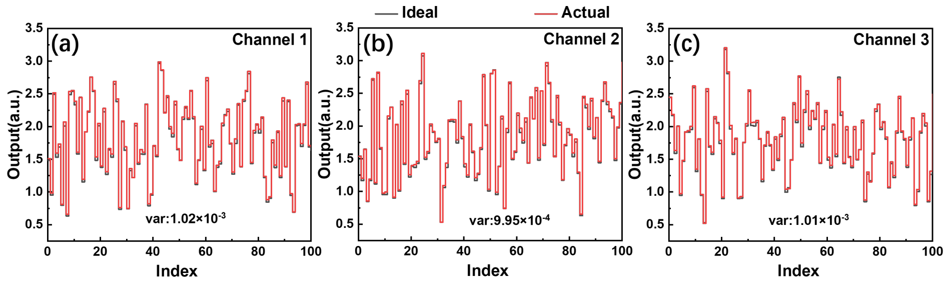

Fig. S8. Comparison of output results for arbitrary inputs with theoretical results after calibration of the MRR array-based system. (a)-(c) correspond to the output cases of the three-channel MAC operations, respectively.

B. Calibration under Nonlinear Response Conditions

Nonlinear responses are primarily induced by power saturation. Previously, nonlinear responses were not considered due to potential damage to the chip

from such power saturation, which typically leads to their exclusion from consideration. In some existing studies, common nonlinear approaches involve performing linear operations on the photonic chip first and then applying additional optoelectronic devices to achieve nonlinear operations[3, 4].

Experiments on various optical signal inputs reveal that the PD response in the optical chip, as shown in Figure S9, does not increase linearly once the input reaches 14 mW. To ensure linear operation, all inputs are typically maintained within the linear response range, thereby securing system linearity and preventing device damage.

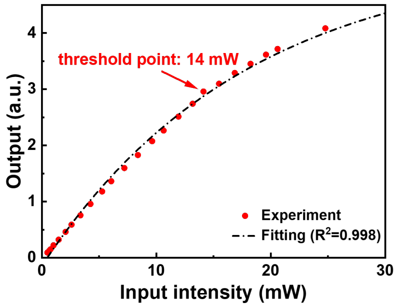

Fig. S9. Response of PD output to variations in input chip power.

To validate the effectiveness of the DRC algorithm while considering nonlinear response in the chip, a Python simulation is executed, under the assumption that the PD exhibits nonlinear response behavior. This response is represented by the fitted curve like the one depicted in Figure S9, which is equivalent to adding the nonlinear component $f_{non}(\cdot)$ to the accumulated operation $F_{chip}(X, W)$ performed on the chip. It is emphasized that the chip achieves linear operation before reaching the PD; thus, the overall operation is expressed as $f_{non}(F(X, W))$.

A basic nonlinear fit, using an exponential decay model, yields the fitting function: $y = 5.384 - 5.528exp(-\frac{x}{17.882})$, achieving an R^2 value of 0.998, which indicates a high-quality fit of the PD response. For simplification, it is assumed that the nonlinear response function of PD is $f_{non}(F_{chip}(X, W)) = 5 - 5exp(-\frac{F_{chip}(X,W)}{4})$. In the context of programming simulations for the micro-ring environment, weights are simplified to the parameters being controlled, and the calibration algorithm remains consistent with **Supplementary Note 2-B**. During the DRC calibration process, target weights are randomly assigned, leading to discrepancies in the outputs of different channels. Figure S10 illustrates the PD outputs across various channels during the calibration process, revealing that each channel completes calibration within approximately 10 iterations.

After calibration, a set of randomly designed input signals is tested for comparison between the actual outputs of the micro-ring array and the ideal outputs. The accumulated results for the three channels, after nonlinear processing through the PD, are compared with theoretical results, as depicted in Figure S11. It was found that the variances of the output errors for the three channels are 1.97×10^{-5}, 3.45×10^{-7} and 1.05×10^{-4}, respectively. These variances are significantly lower than those in the actual system because the process is conducted in a simulation environment, which eliminates the effects of environmental noise and sampling jitter.

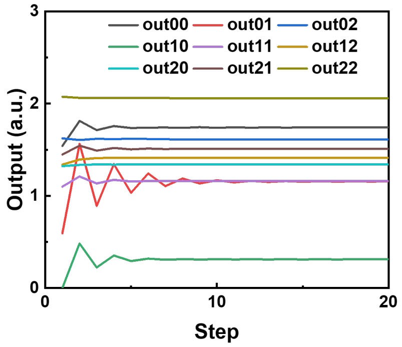

Fig. S10. Variations in output at the PD from different MRRs during the calibration of the simulated 3×3 MRR array.

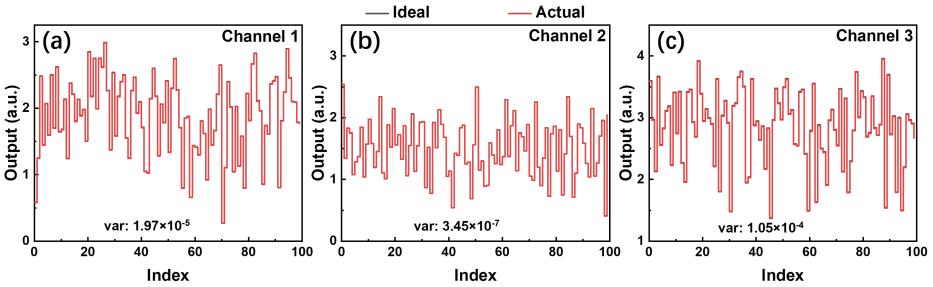

Fig. S11. Comparison of actual outputs with ideal output for arbitrary inputs after calibration of the simulated nonlinear MRR array-based system. (a)-(c) correspond to the output cases of the three-channel calculations, respectively.

Assuming that each micro-ring exhibits a nonlinear response, the DRC method is employed to recalibrate back to linear response conditions. The function to be calibrated can be abstracted as $F_{chip}(f_{non}(X), W)$. For valid solutions to exist, the system must satisfy the condition $x_i \leq \frac{\omega_{max}}{\omega_{exp}} f_{non}(x_i)$, and the function $f_{non}(X)$ must be invertible. This represents a significant boundary for universal calibration methods.

It is further assumed that the nonlinear response function of the micro-ring adheres to an exponential decay model, with the response expressed as $f_{non}(x_i) = 5 - 5exp(-\frac{x}{4})$. The entire micro-ring chip is simulated using Python. Results for the outputs from different micro-rings during the calibration of the simulated 3×3 array are displayed in Figure S12. This system achieves calibration within 10 iterations. Given that the calibration process remains fundamentally consistent regardless of varying complex inputs, this section is not reiterated.

In extending to more complex forms, such as $f_{non1}(F_{chip}(f_{non2}(X), W)$, the goal is to re-calibrate to a standard form of $f_{non1}(F_{chip}(X, W))$. The method referenced earlier may be used, reiterating that the solutions are consistent, thus validation experiments are not included in this section.

6. CALIBRATION PROCESS DETAILS AND CALIBRATION MODELS

Figure S13a extends Fig. 5 presented in the main text, providing a detailed description of the specific process involved in calibrating the multi-wavelength

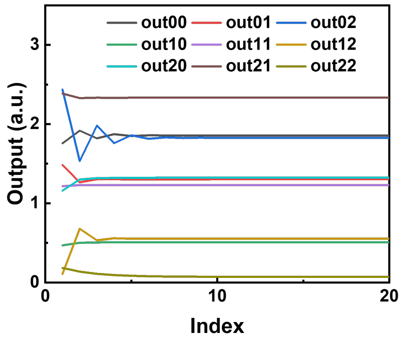

Fig. S12. Variation of output from different MRRs during the calibration process when each MRR in the simulated 3×3 MRR array exhibits nonlinearity.

system using the calibration algorithm.

Figure S13b illustrates the inference and training processes for the DRC model. During the inference phase, the algorithm initially normalizes the output data from the system, resulting in a normalized output state. This normalized state is subsequently inputted into the actor model to deduce the subsequent action. The action derived from the actor model is transformed into specific parameters that are fed back into the system. During the calibration iteration process, if the output reward is higher than in the previous iteration, the data from this calibration will be stored in the replay buffer. Following several iterations, once the output reward surpasses a predefined threshold, it indicates that the target state has been reached, thus concluding the calibration process.

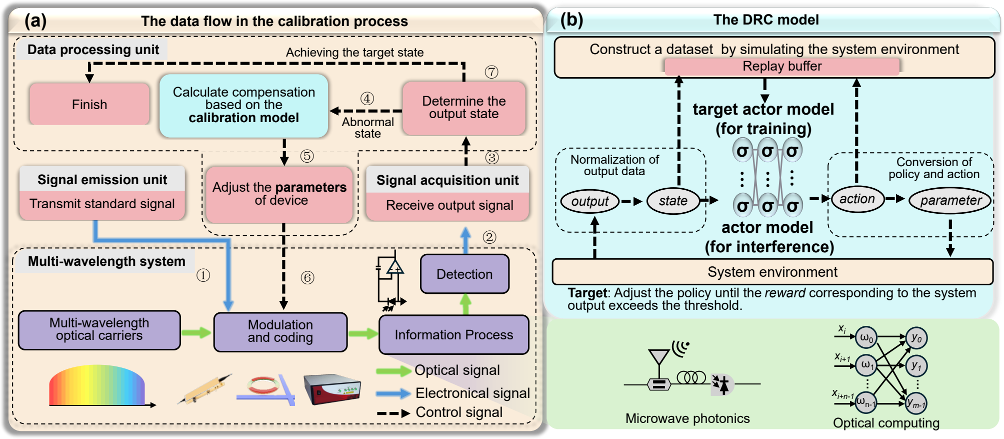

Fig. S13. The detailed architecture of a calibration system utilizing the DRC algorithm for multi-wavelength system. (a) illustrates the detailed flowchart of calibration processing in multi-wavelength system. (b) outlines the training and inference framework for the DRC model. The environment refers to the complete multi-wavelength system.

For the DCF-based and MRR array-based system, in the training phase of the target actor model, the initial approach involves training the DDPG policy model, with the dataset obtained from the actual environment. Despite numerous training iterations, the network fails to converge. This difficulty in convergence can be mainly attributed to two factors. Firstly, the dataset produced through

real-time interaction with the system environment is inadequate, leading to underfitting of the model and poor generalization. Secondly, the randomness of the initial state in the training process leads to significant variations in the policy model in each iteration, complicating the training of the target actor model.

To overcome the challenges of limited sample sizes and significant strategy variations in real-world settings, this study develops a simulated dataset by emulating an multi-wavelength system to train the target actor model. Within an multi-wavelength system, instruments are often required to adjust various wavelength intensities or phase parameters to compensate for the inconsistent wavelength response. By establishing the mapping between system output and the compensation of various wavelengths, a vast array of inputs and outputs can be generated as a simulated dataset for training the target actor model. This approach underpins the training process of the target actor model in both scenarios presented in this study. To improve the adaptability of the target actor model to the multi-wavelength system, multiple sets of data acquired the inference process can also be utilized in the replay buffer for additional training of the target actor model. By the method of soft-update, the new parameters of target actor model is used for further optimization of the actor model. This iterative training approach further refines the calibration strategy and improves the overall performance of the DRC method in real-world applications.

For the MZI array-based system, a one-to-one correspondence between control parameters and output is not established, and the relationship is difficult to describe using a deterministic mathematical equation. Consequently, generating a simulated dataset for training the actor model proves to be challenging. To address this issue, training of the actor model is conducted directly based on the physical system. The training employed the DDPG strategy, utilizing two critic networks (computing Q value for action) and two actor networks (computing actor based on current state) for iterative training. The input and output of the critic network are $[state, action_{cur}]$ and Q-value, respectively. The Q-value is used to represent the evaluation of the action based on the current state. Through continuous optimization of the parameter configuration strategy, the objective is to minimize the iteration period in order to achieve the desired state.

Besides training and inference process of the policy network models, normalizing system output data and designing policy action transformation functions hold significant importance. Experiments have shown that the effectiveness of these two functions directly influences the ability of system to achieve rapid and accurate calibration. These functions are intimately connected to particular calibration tasks, necessitating their adaptation to the specific requirements of the system.

7. FUNDAMENTALS OF CALIBRATION METHODS BASED ON PID, GA, AND SPGD ALGORITHMS

This study involves not only devises and executes the development and execution of a calibration algorithm using DRC but also creation and implementation of calibration algorithms employing PID, GA, and SPGD. Across these algorithms, the reward function assessing the alignment of the system with the desired state remains uniform. Since these details are already provided in the main text, they are not reiterated here. Subsequently, the design principles of the three algorithms are presented sequentially.

First, the PID algorithm is introduced in detail. It can be categorized into two

types: analog PID and digital PID. The analog version relies on continuous-time integration, whereas the digital form employs discrete summation. This study concentrates on the positional algorithm within digital PID, with its fundamental mathematical model outlined below:

$$u_k = k_p \cdot e_k + k_i \cdot \sum_{j=0}^{k} e_j + k_d \cdot (e_k - e_{k-1}) \tag{S12}$$

The pseudocode for the calibration model using the PID algorithm is shown in Algorithm S1.

Algorithm S1. :Calibration model based on PID algorithm

Initial:
Set PID controller parameters k_p, k_i, k_d
Set *target_state* according to the expected value of calibration
Get *current_state* from the environment
Every Iteration:
for $i = 1$ to N **do**
    $error[i] \leftarrow current\_state[i] - target\_state[i]$ ▷ Compute error
    $derivate\_error[i] \leftarrow error[i] - last\_error[i]$ ▷ Compute differential term
    $intergral\_error[i] \leftarrow intergral\_error[i] + error[i]$ ▷ Compute integral term
    $out\_PID[i] \leftarrow k_p \cdot error[i] + k_i \cdot intergral\_error[i] + k_d \cdot derivate\_error[i]$ ▷ Compute $action[i]$
$action \leftarrow out\_PID$
Set *action* to the system
Get *current_state* from the system
Determine if the system has reached the expected state

In the DCF-based system, the PID algorithm employs the primary parameters (k_p, k_i, k_d) set at (0.01, 0.1, 0.05), where N denotes the count of optical comb teeth. For MRR array-based, these parameters are adjust to (0.01, 0.05, 0.5), with N signifying the total MRRs managed in a single operation. The inconsistency in PID configuration parameters across these two systems highlights the difficulty of migrating PID parameters between different types of systems. To evaluate the status of the system, the algorithm calculates the current reward by comparing the *current_state* to predetermined calibration benchmarks. This assessment determines whether the system has achieved the desired state. It is noteworthy that this evaluation method remains consistent throughout the subsequent algorithms discussed in this study.

The SPGD algorithm is widely used in addressing high-dimensional optimization problems, particularly in situations where directly computing the gradient of the objective function is difficult. By estimating the gradient via random sampling, the algorithm updates the actions of system using this approximated gradient. The calibration model that leverages the SPGD algorithm is presented in Algorithm S2.

One of the key advantages of the SPGD algorithm is in eliminating the need for explicit gradient details of the target function, thereby bypassing the high computational costs associated with determining the target state of system. For the two systems mentioned, the parameters α and Δ are set at (0.2, 1) and (0.1, 0.03), respectively.

GA is a widely used heuristic approach that draws inspiration from biological evolution. It operates by simulating natural genetic and evolutionary processes

Algorithm S2. : Calibration model based on SPGD algorithm

Initial:
Set learning rate α
Initialize $action = [0, 0, \cdots, 0]$
Set disturbance intensity Δ
Every Iteration:
$random\_noise \leftarrow [random\_choice([-\Delta, \Delta]), for\ \_\ in\ range(N)]$ ▷ Generate random disturbance
$pos\_action = action + random\_noise$ ▷ positive disturbance
Set pos_action to the system
Get pos_state from the system
$pos\_reward \leftarrow f(pos\_state)$ ▷ $f()$ means the reward function
$neg\_action = action - random\_noise$ ▷ negative disturbance
Set neg_action to the system
Get neg_state from the system
$neg\_reward \leftarrow f(neg\_state)$
$action \leftarrow action + \alpha \cdot (pos\_reward - neg\_reward) \cdot \Delta$ ▷ Update *action*
Set $action$ to the system
Get cur_state from the system
$cur\_reward \leftarrow f(cur\_state)$
Determine if the system has reached the expected state

to identify the optimal solution for a given problem. The development of a calibration technique utilizing GA is outlined in Algorithm S3.

Algorithm S3. :Calibration model based on GA algorithm

Initial:
Generate an initial population $action\_population$, containing N sets of $action$.
Set up the fitness function $fitness\_function$ according to system
Set genetic manipulation parameters: cross probability P_c, variation probability P_m.
while No $action$ taken by the $action\_population$ can achieve the desired state of the system **do**
    Assess the fitness of each $action$ in the $action\_population$
    Selection Operation: Choosing $action$ based on the fitness for offspring production.
    Crossover operation: The selected $action$ are subject to crossover operation according to the probability P_c to generate offspring.
    Mutation operation: perform mutation operation on offspring according to probability P_m.
    Generate a new population $action\_population$.

The role of the $fitness\_function$ in the algorithm is to configure the $action$ into system, and calculate the $reward$ based on its $state$ as the fitness of the $action$. The iteration ends when an $action$ that meets the desired distortion effect is found in a generation of the population.

According to the experimental results, the calibration methods utilizing SPGD and GA are observed to be less efficient. This inefficiency stems from their inherent strong randomness, necessitating a greater number of iterations to finalize the system calibration.

REFERENCES

1. Y. Tong, L. Chan, and H. Tsang, "Fibre dispersion or pulse spectrum measurement using a sampling oscilloscope," Electron. Lett. **33**, 983 (1997).
2. P. Kelkar, F. Coppinger, A. Bhushan, and B. Jalali, "Time-domain optical sensing," Electron. Lett. **35**, 1661 (1999).

3. M. J. Filipovich, Z. Guo, M. Al-Qadasi, *et al.*, "Silicon photonic architecture for training deep neural networks with direct feedback alignment," Optica **9**, 1323 (2022).
4. S. Bandyopadhyay, A. Sludds, S. Krastanov, *et al.*, "Single-chip photonic deep neural network with forward-only training," Nat. Photonics **18**, 1335–1343 (2024).
